# Risky Weapon Carrying Behaviors, Youth Violence, and Substance Use Among Young Black Males in Chicago: A Cross-sectional Analysis

**DOI:** 10.21203/rs.3.rs-4713374/v1

**Published:** 2024-08-14

**Authors:** Chuka N. Emezue, Jessica Bishop-Royse, Tipparat Udmuangpia, Dale Dan-Irabor, Adaobi Anakwe, Wrenetha A. Julion, Niranjan S. Karnik

## Abstract

Objectives . The study evaluates the prevalence of risky weapon-carrying behaviors (WCB) among YBM in Chicago and examines their associations with various forms of direct and vicarious violence—youth violence, community violence, and partner abuse—as well as substance use and substance-related aggression. Methods . We performed Pearson Chi-square tests and multivariable negative binomial regression analysis on cross-sectional data from 266 violence-involved young Black males (YBM) in Chicago. This data was collected using a modified version of the Centers for Disease Control and Prevention's Youth Risk Behavior Survey. Our dependent variable, weapon-carrying behavior, was measured by the frequency of weapon carrying, including items such as guns, knives, and clubs, over the past year. Results . In a sample of 266 YBM (ages 15–24, 99% African American), the mean age was 18.32 ± 3.10 years, and 42.7% had some high school education. The 30-day weapon-carrying incidence was 17.3%, with 19.1% threatening someone with a weapon ≥ 2–3 times in the past year. About one-third engaged in partner violence (30.4%), primarily psychological (36.7%) and physical (28.3%) abuse. Approximately 64.8% experienced some form of violence or aggression in the past year, and 76.4% witnessed community violence. Over 20.8% reported binge drinking, and 43.6% engaged in illicit drug use, with 37.2% participating in or initiating violent acts following alcohol or drug consumption. Negative binomial regression results revealed that exposure to direct and vicarious violence, along with substance use, significantly increased the likelihood of carrying weapons. Specific risk factors such as recent threats or injuries, witnessing violence, involvement in physical altercations, and substance-related aggression significantly predict WCB. Age and relationship dynamics also critically influence these behaviors. Additionally, for each year of age, the risk for WCB increased by 22%. Conclusions . This study identified significant associations between different types of violence, substance use, and risky WCB among YBM in Chicago. The results underscore the need for comprehensive, culturally sensitive, multifaceted interventions addressing both individual and psychosocial factors behind risky WCB. These interventions are crucial for reducing gun violence and improving urban community safety, offering vital data to inform policies and interventions for youth protection in similar environments.

## INTRODUCTION/BACKGROUND

Despite a marked decrease in weapon-carrying among high school-aged boys across all racial, ethnic, and age groups from 1993 to 2019,[1–3] the issue of risky weapon-carrying behaviors (WCB) among Black youth in the US remains a complex public health concern with significant consequences for these young men, their families, and communities. This study examines weapon-carrying behaviors, which refers to the act of carrying different types of weapons in situations with a heightened potential for harm, aggression, or legal repercussions. WCB typically involves carrying firearms, knives, or other weapons in public spaces, schools, or other locations where the possession of weapons poses a significant risk. Firearm-related violence and WCB are notably higher among Black youth,[4] who face disproportionate rates of firearm injuries, homicides, and severe punishments for weapon possession.[3–5] Racially biased stereotypes exacerbate this issue by criminalizing firearm ownership among Black males compared to White males of the same age.[3] Research must consider the unique experiences and rationale for WCB among Black adolescents in high-violence cities—with a paradoxical constellation of evidence-based violence prevention programs.[6]

Recent research has primarily focused on individual risk factors for WCB but has not adequately explored the unique set of interpersonal, community, and societal risk factors that directly influence WCB or the specific motivations underlying it among young Black males (YBM). This study aims to address these gaps by examining the prevalence of WCB and its associations with other types of interpersonal and peer violence, including youth violence, community violence, and partner abuse, as well as substance use among YBM. The ultimate goal is to comprehensively identify and analyze the contributing and interacting factors associated with weapon carrying among YBM to inform intervention and policy design and implementation.

## The Prevalence of Firearm Violence Among US Youth

Firearm-related injuries and fatalities, commonly the result of risky WCB, pose a persistent public health issue, particularly among YBM. In the United States, significant disparities based on race/ethnicity and gender are observed in firearm homicides and violent injuries among adolescents and young adults. Data from 2019 to 2020 reveals a noticeable 39% increase in firearm-related homicides among youths aged 10-24, coinciding with an approximately 15% rise in firearm-related suicides within the same age group.[4] Where firearm homicides were reported, the origin of the firearm remained unknown for up to a third of decedents, suggesting we may be underestimating youth firearm access, especially outside the home.[7] Other studies indicate that even after controlling for sociodemographic factors, past violence victimization, witnessing firearm-involved violence, and witnessing non-firearm-involved violence were both associated with teen firearm carriage (OR: 3.55 [1.86, 6.79] and 4.51 [1.75, 11.6]).[8]

Research that considers gender differences reveals that gun carrying is more common among males (6.8%) than females (1.9%) [9]. In a study of US students in grades 9-12, non-Hispanic Black male students

(10.6%) had the highest rates of gun carrying, followed by Hispanic males (7.2%) and non-Hispanic White males (6.1%).[9] Among females, Hispanic students (3.5%) had higher rates than Black (2.0%) and White students (1.1%).[9] Of those carrying firearms, 46.8% of males and 69.8% of females did so on 1-3 days in the past year, while 42.0% of males and 21.6% of females carried firearms on six or more days during the same period.[9]

Longitudinal studies show a decrease in weapon carrying among all boys in schools without any significant variations in weapon carrying based on race and/or ethnicity.[10] However, male students who reported experiencing violence or feeling unsafe in school were at least twice as likely to bring a weapon to school (Jewett et al., 2021). The same study by Jewett et al. (2021) also found that these negative experiences were more prevalent among boys of color, with rates ranging from 8% to 12%, compared to non-Hispanic white boys, whose rates were between 4% and 5%.[3] New data suggests that non-Hispanic White boys attending schools perceived as safer exhibit a greater propensity to carry guns onto school grounds compared to their non-Hispanic Black/African American or Hispanic peers.[10] In contrast, findings suggest that the incidence of handgun carrying among girls almost doubled from 0.9% to 1.7% between 2002 and 2015 (a figure substantially higher than the increase reported by boys during the same time frame), with the majority of this increase being observed among non-Hispanic White and Hispanic girls.[11] According to a recent measure, approximately one out of every 15 male and one out of every 50 female high school students have reported carrying a gun for non-recreational purposes at least once in the past 12 months.[12]

The existing literature on weapon-carrying among different racial and ethnic groups shows inconsistent findings. Certain studies indicate that Black youth are more likely to carry firearms, whereas non-Black youth tend to carry knives.[13] Conversely, other studies suggest that Black youth are less likely to carry weapons.[14] Some studies indicate that White youth in rural areas are more likely to carry weapons than Black youth in the same settings.[15] Additionally, other studies show that rural American Indian/Alaskan Native youth, lower-income youth, male youth, and older adolescents are more likely to carry handguns.[14] Although the circumstances surrounding WCB may vary by settings, motives, weapon types, and contexts, Black youth are disproportionately affected and bear a significantly higher risk of firearm-related homicides and injuries compared to their White counterparts. This disparity encompasses the broader social and structural determinants of risk factors that influence WCB among Black youth. Examining WCB among YBM requires an intersectional understanding that acknowledges the unique individual, interpersonal, and structural challenges Black youth face in under-resourced communities.

## Risk Factors for Weapon-Carrying Behaviors

Several risk factors are associated with WCB. Studies have shown that gang membership, psychological distress in male adolescents, and exposure to violence, either directly or indirectly, are predictors of gun carrying.[13,16] Earlier research uncovered significant associations between carrying weapons and being male, residing separately from both parents, having a strained relationship with parents, participating in physical fights, vandalizing school property, and the belief that peers are also armed at school.[17] Conversely, ensuing studies identified female gender as a distinguishing factor for students carrying weapons on school grounds versus off school grounds.[18] Moreover, young adolescents who initiated substance use early and engaged in it frequently were more likely to carry guns and other weapons to school after controlling for age, sex, and ethnicity.[19] Importantly, recent findings suggest that risky WCB is also connected to indirect experiences of youth violence, such as observing community violence, regardless of other violence exposures.[8] Risk factors that strengthened in the aftermath of the COVID-19 pandemic are worth noting, as the pandemic intensified risky behaviors and firearm violence. For example, firearm-related homicides among YBM aged 10–24 were 20.6 times higher than among White males of the same age in 2019 but increased to 21.6 in 2020.[2] In 2019, 11.5% to 13.2% of high school students reported carrying a weapon in the past year.[7,20]

At present, our knowledge of the association between these risk factors and various types of youth violence, as well as the distinguishing features of youth who carry weapons compared to those who do not, is limited. Few studies have investigated the association between WCB, various forms of peer and interpersonal violence (youth violence, dating, and partner abuse), vicarious violence (witnessing community violence), and substance use among YBM. Fewer studies have done this utilizing incident rate ratios (IRR) – a statistical metric in epidemiology for comparing rare event rates between two groups (or populations at risk) over time when data are structured to count occurrences and exposure times.

## The Current Study

This cross-sectional study aims to assess the prevalence of risky WCB among YBM and investigates their association with different types of direct and vicarious violence, including youth violence, community violence, and partner abuse, as well as substance use and aggression related to substance use. We examine these factors in a high-violence but program-rich city (i.e., Chicago). Using univariate, bivariate, and multivariable negative binomial regression analysis, we hypothesize a high prevalence of risky WCB among YBM in Chicago due to the likelihood of high levels of exposure to multi-form violence and predict significant links between sociodemographic factors and WCB in this sample. Chicago presents a distinct context for this study due to its high prevalence of firearm-related violence and the numerous intervention programs implemented in response. A cross-sectional study conducted in Chicago between 2013 and 2017, before the pandemic, revealed a rise in firearm homicides among teenagers aged 15-19, with the highest number of cases recorded in 2016.[2] This study found that YBM accounted for 75.6% of all adolescent homicides, increasing to 87.8% during the same period. The rate ratio for YBM compared to other adolescents peaked in 2015 at 19.4 (95% CI, 10.9-34.6).[2]

## Methods

## Study Design and Data Source

A cross-sectional analysis was carried out on a subgroup of 266 youth between the ages of 15 and 24 who participated in a larger study (N=302) to evaluate and pilot-test (*blinded for review*), a culturally adapted, trauma-focused, app-based intervention designed to prevent violence and substance use and minimize the co-occurring risk and consequences among YBM. The protocol of the primary study has been published elsewhere [21]. All stages of the primary study were approved by the Institutional Review Board (IRB) at (*Blinded for Review*). From July 2022 to July 2023, YBM were recruited and enrolled in the larger study through various methods. Recruitment followed the Strategies to Recruit, Engage, and Retain Children in Behavioral Health Risk Factor Studies (REACH) model [22]. Participants were recruited from a male mentoring program (71.5%), the Chicago Black Therapist Network—a website directory of 140 Black therapists in Chicago (3.0%), ResearchMatch (2.3%), flyers (9.5%), direct recruitment by research assistants (1.5%), and social media advertisements (12.2%) to ensure a diverse and representative sample.

Upon expressing interest, YBM completed a sociodemographic enrollment form on REDCap, followed by an eligibility questionnaire to assess their violence and substance use profiles, among other eligibility criteria. This enrollment questionnaire was developed based on the Youth Risk Behavior Surveillance System (YRBS), a survey conducted by the Centers for Disease Control and Prevention (CDC) every two years to gather information about adolescents' risky behaviors starting from 1991. The YRBS survey included self-reported gun and weapon carrying in and outside school settings among high school students in public and private high schools across the United States. We developed a violence and substance use profile comprised of ten questions that assessed constructs related to violence, substance use, and substance use-related violence/aggression. Questions were adjusted as necessary to ensure participants could readily understand and engage with the survey. For example, we rephrased questions related to WCB on school property to be more general, ensuring the questions applied to those not currently attending school—as youth who miss school because of safety concerns report some of the highest prevalence of gun carrying.[12] Additionally, we clarified specific behaviors to eliminate ambiguity. For instance, we provided examples within the question to specify what constituted violent behavior: "In the past year, have you been psychologically aggressive toward a romantic or dating partner (e.g., threatening to hurt them, insulting them, using a hostile or mean tone of voice, threatening them with a weapon, or threatening to hit them or throw something at them)?" We were granted a waiver of parental consent, as the study involved minimal risk. All participants completed informed consent/assent forms using REDCap. In the parent study, participants received incentives after completing each of three phases: the needs assessment, the usability testing of the (*blinded for review*) app, and the pilot study to assess its effectiveness. However, no incentive was provided to complete the enrollment questionnaire.

## Eligibility Criteria

Eligible participants met the following criteria: 1) identified as male, regardless of sexual orientation; 2) self-identified as Black or African American; 3) were between the ages of 15 and 24; 4) Have experienced or witnessed any form of youth violence in their lifetime (for example, physical fighting, dating or relationship violence, school violence, bullying, threats with weapons, and gang-related violence); 5) English fluent; and 6) could provide assent or consent to participate.

### Outcomes Measures

## Dependent Variable

The dependent variable, risky weapon-carrying behavior, was defined as the frequency of weapons carried (number of days) in the previous month (e.g., gun, knife, club). This variable was operationalized as a binary outcome, indicating whether YBM did not carry a weapon (coded as zero) or carried a weapon for at least one day within the previous 30 days in response to this specific question: “During the past 30 days, how many days did you carry a weapon such as a gun, knife, or club?” This question was adapted from the 2023 CDC Youth Risk Behavior Survey (YBRS), which provides crucial insights into youth health behaviors among US students in grades 9 through 12.

## Independent Variables

Sociodemographic variables included age, gender, race/ethnicity, school and employment status, marital and relationship status, and smartphone ownership. Since being non-Hispanic Black was an eligibility criterion for participation in the main study, ethnicity (not race) was considered a covariate.

## Violence outcomes

We analyzed six independent variables, each representing a measure of violence. These variables were: Frequency of physical fighting in the last year; the frequency of being threatened or injured with a weapon within the previous year; situations where someone threatened or harmed them with a weapon; exposure to community violence, such as physical attacks, beatings, stabbings, or shootings (indicated by a Yes/No response); being affected by violence or aggression within the past 12 months (Yes/No). In addition, we assessed psychological aggression towards a romantic or dating partner, which encompassed behaviors such as issuing threats, using derogatory language, adopting a hostile or cruel tone, threatening the partner with a weapon, or making threats of physical harm or throwing objects. Furthermore, we measured physical aggression towards a romantic or dating partner, including slapping, kicking, hitting, punching, or threatening the partner with a weapon.

## Substance use outcomes

We assessed three substance use outcomes: Binge drinking (the number of drinks consumed in a row in the previous 30 days), illicit drug usage (Yes/No to the question “Have you used any medications or drugs other than those required for medical reasons?”), and violence/aggression following drinking/drugs (Yes/No), in response to this question: “Have you been involved in, perpetrated, or committed any sort of violence or aggression after taking any drugs or alcohol?”.

### Data Analysis

All data analyses were conducted using Stata 15.1/SE with the appropriate add-on package (Stata Corp., College Station, TX, USA). Descriptive statistical analysis involved summarizing frequencies and percentages for categorical variables and calculating the mean and standard deviation (SD) for continuous variables. We performed an exponential fit on the frequency of WCB using various demographic characteristics. A series of negative binomial regression analyses identified risk factors associated with WCB using incidence risk ratio (IRR) and 95% confidence interval. We adjusted for selected variables at the multivariate analysis level using various models. Our multivariable analysis began by regressing violence victimization and perpetration measures on risky weapon carrying (Models 1–5). Subsequently, alcohol and drug use measures were added (Models 6–7), followed by measures of aggression and violence towards romantic partners (Models 8–10), demographic characteristics (Model 11), and finally, the full model, including all variables (Model 12). Models and IRR were deemed significant if *p*-values were less than or equal to 0.05.

### Negative Binomial Regression Models

We used negative binomial regression models to analyze our count data, which showed overdispersion (variance exceeding the mean). While we considered other models, such as Poisson regression and zero-inflated negative binomial (ZINB) regression models, we ruled out ZINB because we expected WCB among YBM to be low but not improbable (i.e., true zeros). We used the Akaike Information Criterion (AIC), Bayesian Information Criterion (BIC), and sample size-adjusted BIC to assess the goodness of fit for both Poisson and Negative Binomial models, to identify the optimal number of classes. Generally, the negative Binomial regression results exhibited slightly lower AIC and BIC values, except for the full model where Poisson regression showed lower values. The Negative Binomial model more accurately model count data where Poisson regression may fail, incorporating an extra term for overdispersion. We converted regression coefficients into IRR to interpret percentage changes in the outcome. An IRR of 1.00 signifies no effect, while IRRs below or above 1.00 indicate percentage decreases or increases in risk, respectively. For instance, an IRR of 0.80 indicates a 20% reduction in weapon carrying, while an IRR of 1.20 indicates a 20% increase. This model enabled a comprehensive analysis of key factors influencing the likelihood of WCB, highlighting the predictive power of specific variables and the nuanced relationships between these factors and WCB.

## RESULTS

### Participant Characteristics

A sample of 302 YBM were recruited. After data cleaning, 224 participants aged 15–24 ($M_{age}$ = 18.32 ± 3.10) were retained. Over 51.8% were aged 15–17. Most were African-American (99%) and had some high school education (42.7%). Most identified as students (65.12%). The cumulative incidence of 30-day weapon carrying among YBM (for at least 2–3 days) was 17.3% (almost five times higher than the national average at 3.5%[4] with 57.5% and 8.8% reporting none or at least one day of weapon carrying in the past month. Participants reported threatening someone with a weapon at least 2–3 times in the previous year (19.1%). Even though the majority identified as 'Single' (65.5%), approximately one-third reported perpetrating partner violence (30.4%), primarily psychological partner abuse (36.7%) and physical partner abuse (28.3%). About 64.8% were impacted by any violence or aggression in the past year, and 76.4% had witnessed community violence. Regarding alcohol consumption, 10.22% of YBM reported consuming 1–2 drinks consecutively within the past 30 days, while 20.8% reported engaging in binge drinking—defined for males as consuming five or more drinks in a single session within a two-hour period. A total of 43.6% YBM admitted to engaging in illegal drug consumption, while 37.2% disclosed their participation in or initiation of violent acts following the consumption of alcohol or narcotics.

### Bivariate Analysis

Tables 1 and 2 present the results of Pearson Chi-square bivariate tests to investigate the demographic characteristics and independent factors that predict WCB in this sample. Table 1 shows significant differences in WCB according to age, educational status, and employment status, but not for race/ethnicity or marital status. In total, WCB was most prevalent among YBM aged 14–17 years (53.12%), those with a high school diploma or less (78.20%), and those who were still students (53.12%).

Table 1
Frequency Table for Demographic Characteristics

| | In the Past 30 Days, How Many Did You Carry a Weapon? | | | | | | |
|---|---|---|---|---|---|---|---|
| | 0 day | 1 day | 2–3 days | 4–5 days | 6 + days | Total | $X^2$ |
| Race/Ethnicity | | | | | | | |
| NH Black | 120 (92.31) | 19 (95.00) | 36 (92.31) | 14 (87.50) | 20 (95.24) | 209 (92.48) | 2.68 |
| Hispanic | 8 (6.15) | 1 (5.00) | 3 (7.69) | 2 (12.50) | 1 (4.76) | 15 (6.64) | |
| NH Multiracial | 2 (1.54) | 0 (0.00) | 0 (0.00) | 0 (0.00) | 0 (0.00) | 0 (0.00) | |
| Age | | | | | | | |
| 14–17 yrs. | 94 (72.87) | 5 (25.00) | 11 (28.21) | 0 (0.00) | 9 (45.00) | 119 (53.12) | 82.56*** |
| 18 yrs. | 11 (8.52) | 1 (5.00) | 1 (2.56) | 0 (0.00) | 1 (5.00) | 14 (6.25) | |
| 19–21 yrs. | 8 (6.20) | 1 (5.00) | 8 (20.51) | 6 (37.50) | 6 (30.00) | 29 (12.95) | |
| 22–24 yrs. | 16 (12.40) | 13 (65.00) | 19 (48.72) | 10 (62.50) | 4 (20.00) | 62 (27.68) | |
| Education | | | | | | | |
| HS Diploma or Less | 105 (88.98) | 12 (60.00) | 25 (64.10) | 9 (56.25) | 14 (77.78) | 165 (78.20) | 21.00*** |
| Any College | 13 (11.02) | 8 (40.00) | 14 (35.90) | 7 (43.74) | 4 (22.22) | 46 (21.80) | |
| Marital Status | | | | | | | |
| Single | 91 (70.00) | 13 (65.00) | 23 (58.97) | 7 (43.75) | 14 (66.67) | 148 (65.49) | 10.23 |
| Partnered (Dating, Married, Hooking Up, Common-Law) | 38 (29.23) | 6 (30.00) | 16 (41.03) | 9 (56.25) | 7 (33.33) | 76 (33.63) | |
| No Longer Partnered (Divorced, Separated, Widowed) | 1 (0.77) | 1 (5.00) | 0 (0.00) | 0 (0.00) | 0 (0.00) | 2 (0.88) | |
| Employment Status | | | | | | | |
| Full-Time (35 + hrs/week) | 5 (3.88) | 1 (5.00) | 6 (15.79) | 2 (12.50) | 4 (19.05) | 18 (8.04) | 41.28*** |

| In the Past 30 Days, How Many Did You Carry a Weapon? | | | | | | |
|---|---|---|---|---|---|---|
| Part-Time (less than 35 hrs/week) | 17 (13.18) | 11 (55.00) | 13 (34.21) | 6 (37.50) | 3 (14.29) | 50 (22.32) |
| Student | 84 (65.12) | 7 (35.00) | 15 (39.47) | 4 (25.00) | 9 (42.86) | 119 (53.12) |
| Laid Off, Unemployed, Disabled, Homemaker, Other | 23 (17.83) | 1 (5.00) | 4 (10.53) | 4 (25.00) | 5 (23.81) | 37 (16.52) |
| Note: ***P ≤ 0.001, **P ≤ 0.01, *P ≤ 0.05 | | | | | | |

Table 2
Frequency Table for other variables

| | In the Past 30 Days, How Many Did You Carry a Weapon? | | | | | | |
|---|---|---|---|---|---|---|---|
| | 0 day | 1 day | 2–3 days | 4–5 days | 6 + days | Total | $X^2$ |
| Largest Number of Drinks You’ve Had in a Row in Last 30 days | | | | | | | |
| Did not drink alcohol in last 30 days | 106 (82.17) | 3 (15.00) | 12 (30.77) | 1 (6.25) | 10 (47.62) | 132 (58.67) | 132.20*** |
| 1–2 drinks | 10 (7.75) | 6 (30.00) | 4 (10.26) | 1 (6.25) | 2 (9.52) | 23 (10.22) | |
| 3 drinks | 5 (3.88) | 3 (15.00) | 2 (5.13) | 0 (0.00) | 3 (14.29) | 13 (5.78) | |
| 4 drinks | 3 (2.33) | 2 (10.00) | 3 (7.69) | 2 (12.50) | 0 (0.00) | 10 (4.44) | |
| 5 drinks | 4 (3.10) | 1 (5.00) | 4 (10.26) | 3 (18.75) | 0 (0.00) | 12 (5.22) | |
| 6–7 drinks | 1 (0.78) | 2 (10.00) | 6 (15.38) | 4 (25.00) | 1 (4.76) | 14 (6.22) | |
| 8–9 drinks | 0 (0.00) | 0 (0.00) | 4 (10.26) | 4 (25.00) | 1 (4.76) | 9 (4.00) | |
| 10 + drinks | 0 (0.00) | 3 (15.00) | 4 (10.26) | 1 (6.25) | 4 (19.05) | 12 (5.33) | |
| Drug Use | | | | | | | |
| No | 100 (76.92) | 6 (30.00) | 10 (26.32) | 1 (6.25) | 10 (47.62) | 127 (56.44) | 58.96*** |
| Yes | 30 (23.08) | 14 (70.00) | 28 (73.68) | 15 (93.75) | 11 (52.38) | 98 (43.56) | |
| Ever involved with or committed violence after drinking or using drugs? | | | | | | | |
| No | 111 (85.38) | 6 (30.00) | 14 (35.90) | 2 (12.50) | 9 (42.86) | 142 (62.83) | 70.60*** |
| Yes | 19 (14.62) | 14 (70.00) | 25 (64.10) | 14 (87.50) | 12 (57.14) | 84 (37.17) | |

| In the Past 30 Days, How Many Did You Carry a Weapon? | | | | | | | |
|---|---|---|---|---|---|---|---|
| Psychologically aggressive/violent to romantic partner in past year | | | | | | | |
| No | 117 (90.00) | 6 (30.00) | 10 (25.64) | 2 (12.50) | 8 (38.10) | 143 (63.27) | 96.74*** |
| Yes | 13(10.00) | 14 (70.00) | 29 (74.36) | 14 (87.50) | 13 (61.90) | 83 (36.73) | |
| Physically aggressive/violent to romantic partner in past year | | | | | | | |
| No | 122 (93.85) | 9 (45.00) | 16 (41.03) | 2 (12.50) | 13 (61.90) | 162 (71.68) | 85.13*** |
| Yes | 8 (6.15) | 11 (55.00) | 23 (58.97) | 14 (87.50) | 8 (38.10) | 64 (28.32) | |
| Impacted by any violence or aggression in past 12m | | | | | | | |
| No | 76 (58.46) | 0 (0.00) | 2 (5.13) | 0 (0.00) | 1 (4.76) | 79 (35.11) | 73.93*** |
| Yes | 54 (41.54) | 19 (100.00) | 37 (94.87) | 16 (100.00) | 20 (95.24) | 146 (64.89) | |
| Ever seen someone physically attacked? | | | | | | | |
| No | 48 (36.92) | 1 (5.00) | 2 (5.13) | 1 (6.25) | 2 (9.52) | 54 (23.89) | 28.74*** |
| Yes | 82 (63.08) | 19 (95.00) | 37 (94.87) | 15 (93.75) | 19 (90.48) | 172 (76.11) | |
| Note: ***P ≤ 0.001, *P ≤ 0.05 | | | | | | | |

Table 1

There were significant differences in WCB according to engagement in a variety of independent risk factors, including binge drinking, illicit drug use, involvement with violence after drug/alcohol use, psychological/physical dating aggression towards romantic partners, as well as those who reported being impacted by violence/aggression in past 12 months or who had seen someone physically attacked in their neighborhood. Over half of the sample reported no alcohol consumption in the previous 30 days (58.67%). About 43% of study participants reported illicit drug use (43.56%). Just over a third of the sample (37.17%) reported engaging in violence or aggression after drug/alcohol use.

Table 2

About one-third and one-fourth of participants reported aggression (psychological or physical) towards their romantic partners (36.73% and 28.32%. respectively). Nearly two-thirds of the sample indicated that they had been impacted by violence in the past 12 months (64.89%). Finally, over three-fourths of study participants had seen someone physically attacked.

## Negative Binomial Regression Analysis

Negative binomial regression results are shown below in Table 3. Model 1 (M1) shows that increases in the number of times in the previous month the participant had been threatened or injured with a weapon was associated with an increased risk of weapon carrying by about 40% (IRR 1.550). YBM who were impacted by violence/aggression in the past 12 months had risk ratios 15 times higher (IRR 14.94) than those not impacted by violence (M2).

Table 3

In M4, participants who had witnessed physical violence in their communities had weapon-carrying rate ratios four times higher than those who had not (IRR 4.199). Increases in the number of fights among YBM reported in the previous 12 months was associated with 38% increased incidence (IRR 1.378) in weapon carrying compared to those who reported no fights during that period (M5).

M6 and M7 show participants' alcohol and drug use associated with WCB. In M6, increases in the number of drinks consumed in a row was associated with 27% increased incidence of weapon carrying compared to YBM, who did drink alcohol in the past 30 days (IRR 1.270). YBM who had engaged in illicit drug use in the past year were nearly 3x more likely to carry a weapon than those who had not (IRR 2.986). M8 shows that YBM who had engaged in aggression/violence after drug or alcohol use had weapon-carrying incidence ratios that were over three times larger than those who had not engaged in aggression/violence while under the influence of drugs/alcohol.

Psychological and physical aggression/violence toward a romantic partner were also related to increased incidence of weapon carrying in this sample (M9 and M10). Participants who reported psychological violence/aggression toward a romantic partner in the previous year were nearly 4.5 times as likely to carry a weapon as those who had not been (IRR 4.46). Those who had been physically aggressive toward their partner (M9) had triple the incidence of weapon carrying than participants who had not been physically aggressive to their partner (IRR 3.349).

M12 included demographic characteristics. Every additional year of age increased the risk of weapon carrying by 22% (IRR 1.220). Additionally, employment status was related to incidence risk ratios for weapon carrying; that is, those who worked full-time were more likely than those who were students, not working (due to being disabled, laid off, etc.), or working part-time (IRR 0.490, IRR 0.486, and IRR 0.502, respectively) to engage in WCB.

The full model (M13), which includes controls for demographic characteristics, showed persistent associations for several variables in the presence of covariates. Specifically, YBM reporting a higher number of incidents of being threatened or injured with a weapon were more likely to report carrying a weapon than participants who had not (IRR 1.146). YBM reporting being impacted by any violence or aggression were nearly eight times more likely to carry a weapon than participants who had not (IRR 7.821). Additionally, the number of reported physical fights in the past year was associated with excess risk ratios; participants who reported being in one fight had nearly 11% increased incidence risk than those who reported zero fights during this time (IRR 1.105). Fit statistics indicate the best fitting model to be the fully adjusted model (M13), where the AIC was 463.70.

## Discussion

This study further expands on previous research by using more current data, focusing on YBM in high-violence settings, and examining the impact of substance use and substance-related violence and aggression on weapon-carrying behaviors. To the best of our knowledge, this is the most up-to-date and comprehensive analysis of the relationship between WCB among YBM (ages 15–24) and other forms of direct and vicarious violence and substance use. Our research indicates that particular forms of violence exposure, such as community and peer violence, as well as how exposure occurs, including direct and indirect exposure, are crucial factors in predicting WCB among YBM. These findings highlight the need for a theoretical framework/model that acknowledges the complexity of WCB in underserved and under-resourced minority youth populations. This model will be vital for devising effective culturally informed and risk-sensitive interventions to prevent youth violence and will better help us understand culture- and context-informed ‘mechanisms of action’ of new and existing interventions aimed at reducing firearm homicides and injuries—an area of deficit in the current literature.

Overall, our negative binomial regression results consistently showed that increased weapon carrying was associated with higher exposure to youth violence, community violence, partner abuse, and co-occurring substance use, regardless of the model specifications— underscoring the critical public health issue of WCB among urban youth. YBM who faced threats or injuries in the past month were 40% more likely to carry weapons (IRR 1.550). Those vicariously affected by community violence or aggression in the past year had a 15-fold increase in weapon carrying. Witnessing violence in their community was linked to a fourfold rise in weapon carrying (IRR 4.199). Participants who reported fights in the past 12 months had a 38% higher incidence of weapon carrying. These findings suggest that personal safety concerns and a heightened perception of threat may drive WCB among these young men, supporting theories that emphasize the role of environmental, racial, and economic stressors in shaping behavioral responses.

Alcohol and illicit drug use were also significant factors; each additional consecutive drink increased WCB by 27%, and illicit drug use nearly tripled this likelihood (IRR 2.986)—corroborating studies with nationally representative samples, showing that substance use remains associated with higher prevalence of gun carrying.[12] Aggression or violence following substance use was associated with a threefold increase in weapon carrying, underscoring the need for integrated substance use interventions within violence prevention programs aimed at this demographic. Other studies indicate marijuana, but not alcohol use, was uniquely associated with firearm conflicts.[23] Our fully adjusted model confirmed these patterns, showing the continuous impact of general substance use and WCB. Integrated substance use programs that address the underlying causes of firearm violence are mission-critical. These programs should include trauma-informed counseling and culturally responsive rehabilitation services tailored to the needs of YBM—such as male mentoring programs, educational and vocational training programs to improve economic mobility, health education workshops addressing both physical and mental health needs, sports and recreation programs, and legal counseling and advocacy to help YBM navigate the justice system, understand their rights, and receive fair treatment. Given the reality of funding constraints, community-based programs could operate during peak periods of firearm violence, like weekends and summer months.

Psychological and physical aggression towards a romantic partner also elevated the incidence of WCB (IRR 4.46 and IRR 3.349, respectively). However, this association is likely bidirectional. The association between dating violence and WCB has received scant attention in the literature.[24] Co-occurring with dating violence, WCB may indicate broader issues related to conflict resolution, communication, and emotional regulation skill deficits among YBM. This suggests that evidence-based dating violence interventions, such as Safe Dates[25] or Dating Matters®[26], could be further beneficial when combined with discussions about weapon carrying and firearm safety in the context of dating relationship. Sociodemographically, employment status also influenced WCB risk; full-time workers were more likely to carry weapons compared to students or part-time workers, possibly because full-time employment provided YBM with the disposable income and legal eligibility to purchase firearms. The risk of WCB also increased by 22% for each additional year of age (IRR 1.220), potentially connected to accumulating daily negative experiences and stressors or greater access to weapons as these young men reach the legal age for firearm purchases—which varies by state and type of firearm. This age-related trend in WCB underscores the significance of employing early and primary interventions for young boys who have yet to be fully subjected to firearm violence and other direct or vicarious forms of peer and interpersonal violence.

Although few studies have examined the complexity of factors underlying WCB in high-risk environments, our findings align with previous research that has shown an increased likelihood of gun carrying among males (IRR 1.41, CI 1.27–1.58), non-Hispanic Black youth (IRR 1.26, CI 1.07–1.48), and youth with high scores on the violence index—those at the highest risk levels.[27] In addition, Kemal et al.[27] found that for each additional point on their ad-hoc violence index, there was a 1.74-fold increase in the likelihood of gun carrying among first-year and second-year students in public schools in Chicago, New York City, and Los Angeles. Notably, Chicago's firearm homicide rate is quadruple the national average,[28] as epidemiological data reveal significant mortality and morbidity rates among YBM in Chicago's West, South, and Southwest communities,[29] characterized by high levels of concentrated disinvestment, poverty, unemployment, and public assistance use. One study found a 14-year disparity in life expectancy between neighborhoods just five miles apart in Chicago.[30] Additionally, despite comprising only 15% of the city's population, ten of the 77 neighborhoods account for more than 50% of all shootings in Chicago.[31]

We will be remiss not to mention that the COVID-19 pandemic sparked a significant rise in gun ownership and carrying, fueled by worries about violence and safety.[32–34] This proliferation in firearm ownership was pronounced among prior gun owners and political conservatives, who increasingly viewed guns as a means of protection.[33] Likewise, disparities in state or local gun policy between urban and rural areas, unequal enforcement of gun laws contributed to the rise in gun ownership.[14] These findings collectively underscore the relationship between WCB and socio-political influences in forming attitudes toward gun ownership and carrying. How these policymaking impact WCB among YBM, whether or not they are above or below the legal age of ownership, is a critical point of consideration. Also, crucial are policy response to non-gun weapons in and outside of school settings.

## Limitations and Study Strengths

The study has some limitations that should be acknowledged along with its strengths. It is crucial to recognize that although this study presents a comprehensive overview of WCB among YBM in Chicago, the findings only demonstrate an association and risk ratio and cannot establish causal or directional relationships. Moreover, even though our sample size and response rate were appropriate for detecting statistically significant correlations, these results may not apply to all comparable urban settings or demographic groups.

Additionally, youth who exclusively engaged in hunting or target/sport shooting with firearms or those who carried or fired firearms as gang members were not explicitly included in our analysis. However, our qualitative interviews with this sample suggest firearm use related to past or current gang membership (*citation blinded*). Our survey was designed in a manner similar to the YBRS questionnaire, which we adapted to fulfill our requirements. The YBRS inquired about weapons in general and did not differentiate among specific categories of weapons. Additionally, we did not analyze long-term weapon-carrying patterns to effectively capture time trend effects among YBM—an acknowledged limitation in the field.[15] In addition to considering long-term weapon carrying, evaluating seasonal and episodic weapon carrying is essential. Future research should investigate the frequency of weapon carrying during peak violence times, like nights, weekends, and summer months, and aim to clarify the reasons for gun carrying among YBM, which are currently unknown.

Social desirability and recall biases may have an impact on self-reported WCB in our sample. Studies suggest that males tend to take pride in carrying weapons, which is associated with a desire for power, protection, status, and identity. This is particularly true in high-risk environments where carrying a weapon can symbolize status and power projection.[35] According to Dijkstra et al.[35], adolescent weapon carrying stems from a multifaceted interplay between the appeal of weapon carrying for affiliation, peer influence within friendship networks, and societal factors (e.g., concentrated poverty, neighborhood disadvantage, structural violence, or racism). Additionally, our study faced limitations in capturing the full spectrum of dating violence—factors like gender bias and underreporting affect the perception and investigation of sexual violence among young males, leading to its exclusion from our questionnaire.

The messaging for secondary and tertiary prevention programs that focus on restrictive gun access approaches ("means restriction") and community prevention events (e.g., gun return and buybacks) could be enhanced to offer more effective support for high-risk YBM. Although harm reduction messaging is vital for reducing risky behaviors, it may not be effective for YBM in high-risk communities. These young men become exposed to additional harm and are at risk for retaliatory violence and homicide when they are unable to carry weapons or change their weapon-carrying behaviors, especially if they have a history of perpetrating violence, are involved in gangs [36], or report experiencing police violence. Studies suggest a strong relationship between attitudes towards the police and weapon carrying among Black youth than among non-Black youth [37], highlighting the need for community-based interventions that address underlying risk factors, such as systemic racism and mistrust of law enforcement, and the need for laws that protect YBM from exposure to structural violence and provide safe havens in communities identified as high-risk for firearm-related violence.

Several strengths are worth noting. The insights gained from this study can inform the design and implementation of targeted firearm violence interventions and policy formulations to reflect current realities and effectiveness data. By implementing a comprehensive approach that addresses the intersections of violence exposure, racial trauma, and deficits in behavioral health services, both new and existing programs can be better tailored to meet the specific needs of YBM in high-violence settings. In addition, the broader objective of the study was to develop and pilot (blinded for review), a digital initiative aimed at reducing youth and firearm violence among YBM aged 15–24. The emerging role of mHealth and digital interventions in addressing WCB and firearm violence presents new avenues for addressing gaps in support services, especially in cities with an abundance of prevention programs but low voluntary program uptake. Advocates for leveraging mHealth technology to expand access to services recommend employing Ecological Momentary Assessment (EMA) and Timeline Follow-Back (TLFB) methodologies, such as daily diaries[23] and more intensive longitudinal studies, to thoroughly track daily carriage behaviors.[15] This approach can enhance our understanding of firearm carriage, provide insight into the motivations behind firearm conflicts across diverse populations, and aid in developing future technology-enhanced interventions. Methodologically, employing negative binomial regression allowed for a robust analysis of potential predictors of WCB risk, offering clear advantages over traditional dichotomous multivariable logistic regression by effectively handling overdispersion and the zero-inflated nature of WCB frequency data among YBM.

### Conclusion

In conclusion, firearm violence remains a complex yet preventable public health issue. To date, our analysis is among the most thorough and current examinations of the relationship between weapon-carrying behaviors (WCB) and various forms of violence and substance use among YBM aged 15–24. Our findings underscore the significance of specific types of violence exposure—including community and peer violence—and the modes of this exposure, whether direct or indirect, as critical predictors of WCB in this demographic. These strong correlations between WCB and various forms of violence and substance use underscore the urgent need for comprehensive, culturally sensitive, and multifaceted interventions. Such interventions should aim to reduce WCB and tackle the underlying peer, social, and psychological factors driving these behaviors. Given the rising rates of gun-related homicides, especially in areas with high firearm accessibility, future longitudinal studies on WCB are vital. These studies will be crucial for informing policies and interventions designed to protect youth in similar urban settings.

### Abbreviations

• IRR
• Incidence Risk Ratio
• WCB
Weapon-Carrying Behaviors
• YBM
• Young Black Boys and Men

### Declarations

## Availability of data and material

The datasets used and analyzed in the current study are available through Open Science Framework via this link: https://osf.io/z3sh4/

**Acknowledgement:**

The researchers declare no conflicts of interest or financial disclosures. We thank Dr. Marlon Haywood, Mr. Rickey Layfield, and the Urban Male Network for their invaluable assistance. We also appreciate the young men who participated in this study and the reviewers and journal editors for their insightful feedback.

### Funding Information:

This project received financial assistance from several sources, including the Chicago Chronic Condition Equity Network (C3EN), supported by the National Institute on Minority Health and Health Disparities (NIMHD; Grant Number 5P50MD017349-02). Furthermore, the Institute for Translational Medicine (ITM) provided additional funding obtained through the NIH/NCATS-funded Pilot Translational and Clinical and Translational Science Awards under Grant Numbers UL1TR002389, KL2TR002387, and TL1TR00238. Lastly, the Rush-BMO Institute for Health Equity (RBIHE) Scholars Program at Rush University also contributed to the funding of this study. This paper precedes a series of studies focused on the development, adaptation, and pilot testing of BrotherlyACT. The funding source did not influence the study's design, conduct, data collection, management, analysis, interpretation, manuscript preparation, review, approval, or the decision to submit the manuscript for publication.

### Contributions

All authors (CE, NK, BS, AA, JRB, DDI, APF, AD, QL, WJ) contributed to the study conception, design, supervision, writing—review and editing. Material preparation, data collection, validation, and analysis were performed by CE and JBR. The first draft of the manuscript was written by CE, and all other authors commented on previous versions of the manuscript. All authors read and approved the final manuscript.

### Author information

*Authors and Affiliations*

**Department of Women, Children, and Family Nursing, College of Nursing, Rush University Medical Center, Chicago, IL, USA**

Chuka N. Emezue, Jessica C. Bishop-Royse, Wrenetha Julion

**Department of Psychiatry, Institute for Juvenile Research (IJR), University of Illinois Chicago, Chicago, IL, USA**

Niranjan S. Karnik

**Department of Health Management and Policy, Dornsife School of Public Health, Drexel University, Philadelphia, PA, USA**

Adaobi Anakwe

**Praboromarajchanok Institute, Faculty of Nursing, Boromarajonani College of Nursing, Khon Kaen, Thailand.**

Tipparat Udmuangpia

**School of Humanities and Social Sciences, University of Missouri Kansas City - Volker Campus, Kansas City, USA**

Dale Dan-Irabor

**Contributions**

DIS conceptualized and designed the study, carried out the initial analyses, and drafted the initial manuscript. BA drafted the initial manuscript and revised the manuscript. RH, JL, ER, and TRM critically reviewed and revised the manuscript. All authors have read and approved the final manuscript.

**Ethics declarations**

*Ethics approval and consent to participate*

This study did not meet the definition of human subjects research and therefore was not subject to IRB review. However, IRB approval and written informed consent was obtained from all participants in the main study. We certify that the study was performed in accordance with the ethical standards as laid down in the 1964 Declaration of Helsinki and its later amendments or comparable ethical standards.

**Consent for publication**

Not applicable.

**Competing interests**

The authors declare that they have no competing interests.

## Table 3

Table 3 is available in the Supplementary Files section.

## Supplementary Files

This is a list of supplementary files associated with this preprint. Click to download.

- TABLE3.docx
